# Molecular Insights and Prognosis Associated With RBM8A in Glioblastoma

**DOI:** 10.3389/fmolb.2022.876603

**Published:** 2022-04-29

**Authors:** Lei Wei, Chun Zou, Liechun Chen, Yan Lin, Lucong Liang, Beiquan Hu, Yingwei Mao, Donghua Zou

**Affiliations:** ^1^ Department of Neurology, The Fifth Affiliated Hospital of Guangxi Medical University, Nanning, China; ^2^ Department of Neurology, The Second Affiliated Hospital of Guangxi Medical University, Nanning, China; ^3^ Department of Medical Oncology, Guangxi Medical University Cancer Hospital, Nanning, China; ^4^ Department of Neurosurgery, The Fifth Affiliated Hospital of Guangxi Medical University, Nanning, China; ^5^ Department of Biology, Pennsylvania State University, University Park, PA, United States

**Keywords:** glioblastoma, RBM8A, cox regression, overall survival, candidate mRNAs

## Abstract

**Background:** Glioblastoma (GBM) is the most invasive brain tumors, and it is associated with high rates of recurrence and mortality. The purpose of this study was to investigate the expression of RBM8A in GBM and the potential influence of its expression on the disease.

**Methods:** Levels of RBM8A mRNA in GBM patients and controls were examined in The Cancer Genome Atlas (TCGA), GSE16011 and GSE90604 databases. GBM samples in TCGA were divided into RBM8A^high^ and RBM8A^low^ groups. Differentially expressed genes (DEGs) between GBM patients and controls were identified, as were DEGs between RBM8A^high^ and RBM8A^low^ groups. DEGs common to both of these comparisons were analyzed for coexpression and regression analyses. In addition, we identified potential effects of RBM8A on competing endogenous RNAs, immune cell infiltration, methylation modifications, and somatic mutations.

**Results:** RBM8A is expressed at significantly higher levels in GBM than control samples, and its level correlates with tumor purity. We identified a total of 488 mRNAs that differed between GBM and controls as well as between RBM8A^high^ and RBM8A^low^ groups, which enrichment analysis revealed to be associated mainly with neuroblast proliferation, and T cell immune responses. We identified 174 mRNAs that gave areas under the receiver operating characteristic curve >0.7 among coexpression module genes, of which 13 were significantly associated with overall survival of GBM patients. We integrated 11 candidate mRNAs through LASSO algorithm, then nomogram, risk score, and decision curve analyses were analyzed. We found that RBM8A may compete with DLEU1 for binding to miR-128-1-5p, and aberrant RBM8A expression was associations with tumor infiltration by immune cells. Some mRNAs associated with GBM prognosis also appear to be methylated or mutated.

**Conclusions:** Our study strongly links RBM8A expression to GBM pathobiology and patient prognosis. The candidate mRNAs identified here may lead to therapeutic targets against the disease.

## Background

Glioblastoma (GBM), the most common primary brain tumor in adults, grows rapidly, invades diffusely, and resists treatment (Zhao et al., 2019). Despite recent advances in multimodal treatment combining maximal surgical resection with postoperative adjuvant chemotherapy, the 5-year survival rate of patients is less than 5% (Novak et al., 2020; Lau et al., 2014). This highlights the urgent need for more effective targeted therapies.

Developing such therapies depends on elucidation of the molecules and genes that control GBM growth and on the alterations in the immune system that allow the disease to progress. Standing in the way is the heterogeneity of GBM genes and molecules (Parker et al., 2015): GBM shows substantial genomic instability, proliferation, angiogenesis, anti-apoptotic capacity, and propensity for necrosis (Fathi Kazerooni et al., 2020). This causes significant morbidity, recurrence, and poor response to treatment.

The advent of gene expression has provided new insights into the molecular and mutational features of GBM (Young et al., 2018; Xu et al., 2019). Such profiling in other cancers has implicated RNA-binding motif protein 8A (RBM8A), a member of the RNA-binding motif protein (RBM) family (Tatsuno and Ishigaki, 2018). RBM8A is aberrantly expressed in colon adenocarcinoma, gastric cancer, and hepatocellular carcinoma, and it is an important prognostic marker and therapeutic target (Lin et al., 2019; Liang et al., 2020; Lv and Cheng, 2020; Xie et al., 2021). In addition, our previous study also showed that abnormal expression of RBM8A affects neural cell development (Zou et al., 2015; Zou et al., 2019; McSweeney et al., 2020). Loss of RBM8A function influences multiple genes associated with neurodegenerative or neuropsychiatric disorders, and the protein is essential for cortical neural progenitor proliferation and differentiation (Zou et al., 2015). RBM8A also regulates the expression of the proto-oncogene p53, altering sensitivity to DNA damage and thereby influencing tumor development (Lu et al., 2017). In normal mammalian development and physiology, RBM8A is involved in cell differentiation, apoptosis, RNA splicing, and cell cycle regulation (Ishigaki et al., 2013; Zhang et al., 2017; McSweeney et al., 2020).

This led us to wonder whether RBM8A might be involved in GBM. Therefore, we explored the expression profile of RBM8A in GBM patients. Using a bioinformatics approach, we analyzed genes, genetic mutations, methylation modifications and molecular functions associated with alterations in RBM8A expression in GBM tissues and the T98G cell line of GBM. Our findings may provide novel targets and strategies for GBM prognosis and treatment.

## Materials and Methods

### Data Collection

Data on mRNA sequencing (mRNAseq) and long non-coding RNAs (lncRNAs) from 145 GBM patients and 5 controls was obtained from The Cancer Genome Atlas (TCGA) (https://portal.gdc.cancer.gov/). Gene expression profiles were also acquired from GSE16011 and GSE90604 in the Gene Expression Omnibus (GEO) database (https://www.ncbi.nlm.nih.gov/geo/). In GSE16011, mRNAs and lncRNAs were profiled in 276 GBM samples and 8 control samples; in GSE90604, data came from 16 GBM samples and 7 healthy brain samples, which included mRNA expression profiles in GSE90598 and microRNA (miRNA) expression profiles in GSE90603. The Oncomine database was used to analyze RBM8A mRNA expression in human tumors.

### RNA Sequencing

The RBM8A-overexpressing T98G cells were constructed using lentiviral expression plasmids and validated using quantitative real-time polymerase chain reaction (qRT-PCR) and western blots, which descripted in our previous work (Lin et al., 2021). Total RNA was isolated from RBM8A-overexpressing T98G cells and control T98G cells using TRIzol (Thermo Scientific, Uppsala, Sweden) and purified using the RNeasy kit (QIAGEN). RNA-Seq libraries were constructed using the TruSeq Stranded mRNA-Seq Library Preparation Kit (Illumina). Samples were sequenced using the Illumina NovaSeq system, generating paired-end reads of 150 bp. Raw sequence reads were converted into fragments per exon kilobase per million mapped reads (FPKM) in order to quantify gene expression.

### Difference Analysis

The expression of RBM8A was compared between GBM and normal samples in TCGA, GSE16011, and GSE90598. The “DEseq2” package in R (Love et al., 2014) was used to identify mRNAs and lncRNAs whose expression differed between GBM and controls in TCGA, mRNAs whose expression differed between RBM8A^high^ and RBM8A^low^ GBM samples in TCGA, and mRNAs whose expression differed between RBM8A-overexpressing T98G cells and control T98G cells. The “limma” package in R (Ritchie et al., 2015) was used to identify mRNAs and lncRNAs whose expression differed between GBM and controls in GSE16011, mRNAs whose expression differed between GBM and controls in GSE90598, and miRNAs whose expression differed between GBM and controls in GSE90603.

In all analyses, differentially expressed mRNAs (DEmRs), lncRNAs (DElncRs) and miRNAs (DEmiRs) were defined as those whose expression difference was associated with *p* < 0.05.

### Enrichment of DEmRs in Functions and Pathways

The “clusterProfiler” package in R (Yu et al., 2012) was used to analyze DEmRs for enrichment in Gene Ontology (GO) biological processes (BPs) and in Kyoto Encyclopedia of Genes and Genomes (KEGG) pathways. *p* < 0.05 was defined as significant enrichment. Gene set variation analysis (GSVA) was performed using the “GSVA” package in R (Hänzelmann et al., 2013) to order to display enrichment results. The clusterProfiler package was also used to perform gene set enrichment analysis (GSEA) (Subramanian et al., 2005).

### Target Prediction

The software lncBase 3 (https://diana.e-ce.uth.gr/lncbasev3) was used to predict the target miRNAs of lncRNAs that were significantly associated with overall survival of GBM patients. The target mRNAs for miRNAs were predicted using miRWalk and TargetScan databases. A network of competing endogenous RNAs (ceRNAs) was constructed based on the predicted negative regulation of miRNAs.

### Construction of a Coexpression Network

Multiscale embedded gene coexpression network analysis (MEGENA) was performed using the “MEGENA” package in R (Song and Zhang, 2015). MEGENA builds multiscale networks of potential gene-gene interactions. The default parameters were used to calculate a planar filtered network (PFN) from gene expression correlations in MEGENA.

### Construction of a Model to Predict Overall Survival of GBM Patients

Cox regression was used to identify DElncRs and DEmRs associated with overall survival of GBM patients. Survival curves were generated using the “survival” package in R. The “glmnet” package in R (Friedman et al., 2010) was used to integrate potentially prognostic mRNAs into a binomial least absolute shrinkage and selection operator regression (LASSO) model. During LASSO regression, we retained potential predictors with non-zero coefficients and lead to candidate mRNAs. Areas under receiver operating characteristic curves (AUCs) were calculated using the “pROC” package in R (Robin et al., 2011). A prognostic nomogram was constructed *via* Cox regression to predict 3- and 5-year overall survival of GBM patients in TCGA.

### Construction of a Risk Score-Based Prognostic Model

Candidate mRNAs were selected to construct a prognosis model based on a risk score. GBM patients were divided into low- or high-risk groups based on median risk score, and the corresponding Kaplan-Meier curves were generated and compared using the “survival ROC” package in R.

### Analysis of Drug Sensitivity

Drug sensitivity of DEmRs in RBM8A^high^ and RBM8A^low^ GBM samples was predicted (in terms of IC_50_) based on the Genomics of Drug Sensitivity in Cancer database (www.cancerRxgene.org).

### Immune Cell Infiltration

Levels of tumor infiltration by immune cells were evaluated using CIBERSORT (https://cibersort.stanford.edu/) and single sample gene set enrichment analysis (ssGSEA) in the “GSVA” package in R. Immune cells expressed as 0 were excluded from the analysis. Differences in levels of immune cell infiltration were calculated between GBM and control samples, as well as between RBM8A^high^ and RBM8A^low^ samples, using the “limma” package in R. We also evaluated potential correlations between candidate mRNAs and immune cells using Pearson correlation analysis. Results associated with *p* < 0.05 were considered statistically significant.

### DNA Methylation and Somatic Mutations

The “cAMP” package and “maftools” package in R (Mayakonda et al., 2018) were used to identify differences in methylation or somatic mutations, respectively, between GBM and control samples in TCGA.

## Results

### High Expression of RBM8A in GBM

**FIGURE 1:**
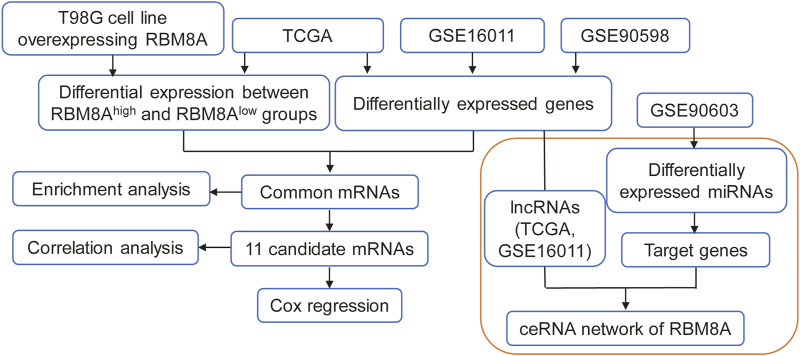
The flow diagram of this study. ceRNA, competing endogenous RNAs; lncRNA, long non-coding RNAs; LASSO, least absolute shrinkage and selection operator regression; MEGENA, multiscale embedded gene coexpression network analysis; ROC, receiver operating characteristic curves; TCGA, The Cancer Genome Atlas.

**FIGURE 2:**
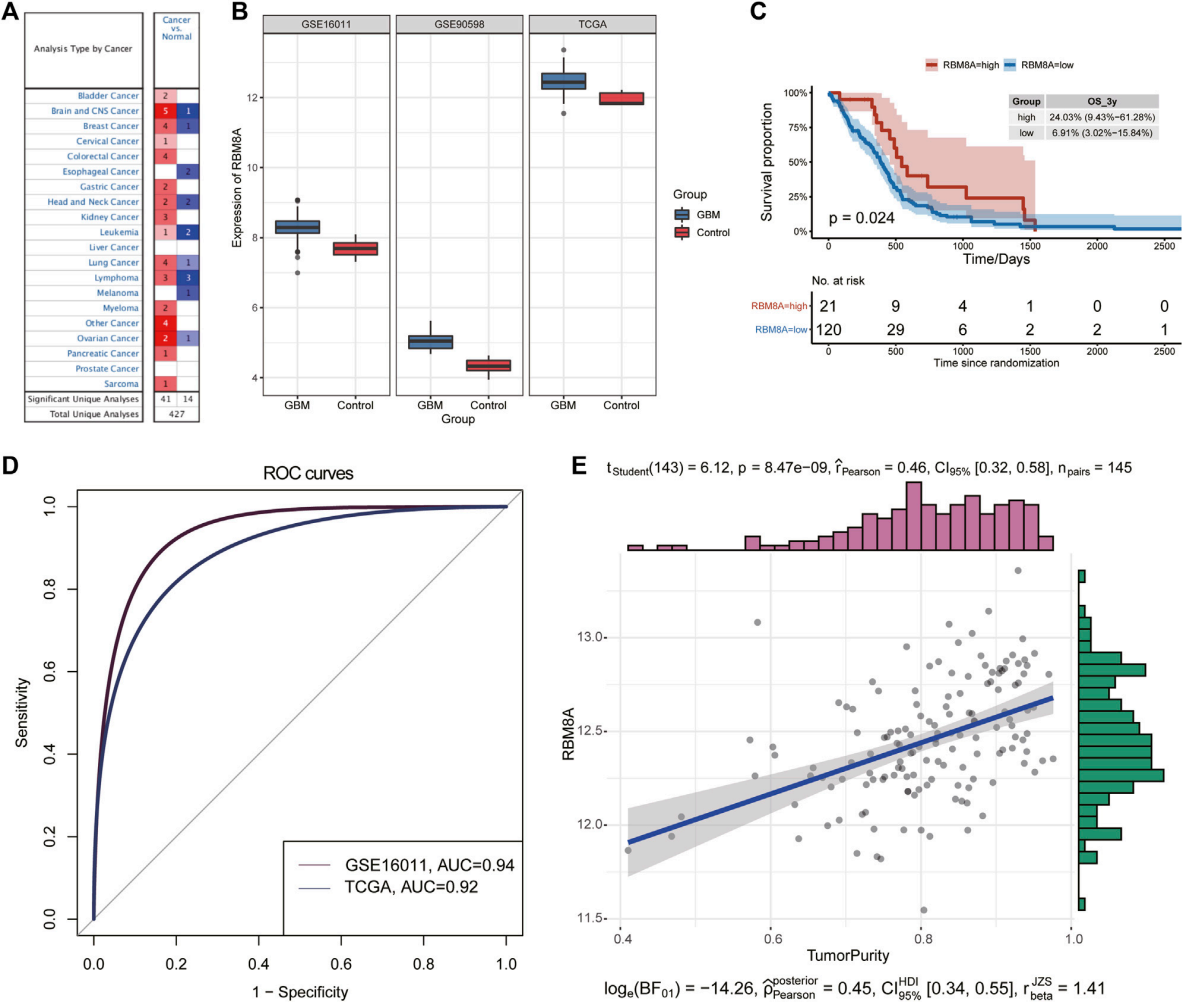
Identification of aberrant RBM8A expression in tumor samples. **(A)** Expression of RBM8A across many cancers. **(B)** Differences in RBM8A expression between GBM and controls in TCGA, GSE16011, and GSE90598. **(C)** Kaplan-Meier curves for GBM samples based on optimal gene expression grouping. GBM patients expressing high RBM8A had poor prognosis. **(D)** Receiver operating characteristic curves to assess the ability of RBM8A expression to predict GBM in TCGA, GSE16011, and GSE90598. **(E)** Correlation between RBM8A and tumor purity in TCGA.

The flowchart of this study is shown in Figure 1. First, the mRNA expression of RBM8A in human tumors was analyzed using the Oncomine database. Its expression was found to be higher in cancer tissues than in controls (Figure 2A). To identify the potential role of RBM8A on GBM, we characterized RBM8A expression in three datasets: TCGA, GSE16011, and GSE90598. We found that RBM8A was abundantly expressed in GBM tissues but weakly expressed in normal controls (Figure 2B). Interestingly, we found that GBM patients with high expression of RBM8A had poor overall survival based on the optimal threshold for gene expression grouping (Figure 2C). The receiver operating characteristic curves for RBM8A to predict GBM gave AUCs >0.9 for all three datasets (Figure 2D). These results indicate that RBM8A expression is upregulated in GBM and may contribute to tumorigenesis. Indeed, expression of RBM8A showed a positive correlation with GBM tumor purity in TCGA (Figure 2E).

### Identification of RBM8A-Related mRNAs in GBM

**FIGURE 3:**
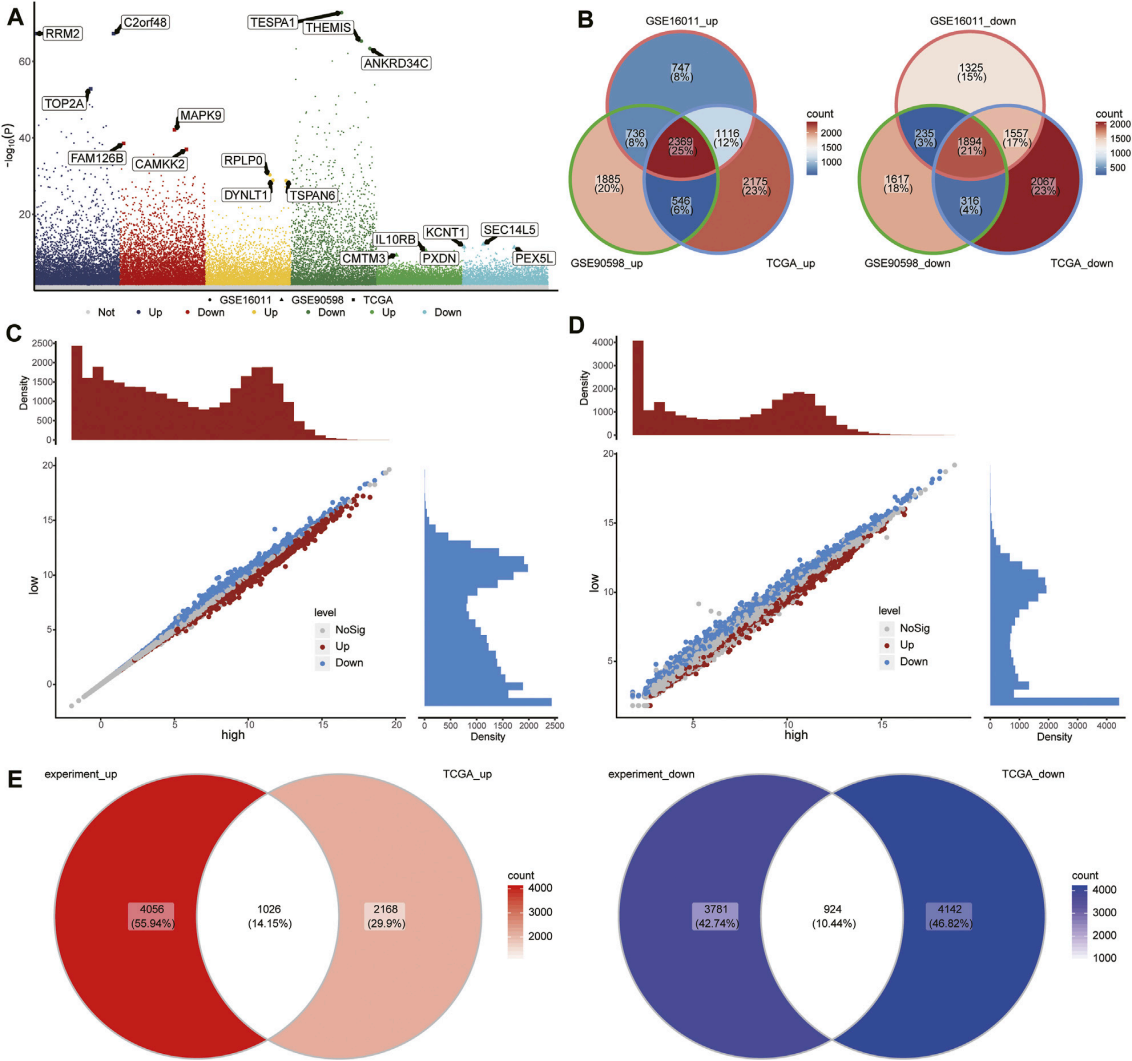
Differentially expressed mRNAs (DEmRs) between the RBM8A^high^ and RBM8A^low^ groups. **(A)** Manhattan plot of DEmRs in TCGA, GSE16011, and GSE90598. The top 3 genes with the largest fold change in differential expression were labeled. **(B)** Intersection of DEmRs up- or downregulated across all three datasets. **(C)** DEmRs between RBM8A-overexpressing T98G cells and control T98G cells. **(D)** DEmRs between RBM8A^high^ and RBM8A^low^ groups in TCGA. **(E)** Overlap between the DEmRs in panels **(C,D)** consider as common mRNAs.

To identify GBM-associated mRNAs, we performed differential analysis of mRNA expression between GBM and controls in TCGA, GSE16011, and GSE90598. We identified 12,040 DEmRs in TCGA, 9,979 DEmRs in GSE16011, and 9,598 DEmRs in GSE90598 (Figure 3A). Among these DEmRs, 2,369 were all upregulated and 1894 were downregulated in GBM (Figure 3B). These mRNAs may be GBM-associated mRNAs.

To begin to elucidate the consequences of aberrant expression of RBM8A in GBM, we transcriptionally profiled RBM8A-overexpressing (OE) T98G cells and control cells, leading us to identify 9,787 DEmRs (Figure 3C). In addition, we divided the GBM samples in TCGA into RBM8A^high^ and RBM8A^low^ groups, and we identified 8,260 DEmRs between the two (Figure 3D), of which 1,024 were upregulated and 924 downregulated in the RBM8A^high^ group (Figure 3E). A total of 488 of the DEmRs between RBM8A^high^ and RBM8A^low^ groups were also DEmRs between GBM and controls. These common mRNAs may be associated with both RBM8A expression and GBM tumorigenesis.

### Biological Functions of RBM8A in GBM

To identify potential roles of RBM8A in GBM, we performed GSEA for GBM and controls. GBM was associated with activation of asthma, p53 signaling, as well as antigen processing and presentation (Supplementary Figure S1A). Conversely, the disease was associated with suppression of the cAMP signaling pathway, calcium signaling pathway, and retrograde endocannabinoid signaling. We also performed GSEA for RBM8A^high^ and RBM8A^low^ groups. RBM8A^high^ status was associated with activation of hedgehog signaling, Notch signaling, and Fanconi anemia, but with suppression of JAK−STAT signaling, NOD-like receptor signaling, and cytokine-cytokine receptor interaction (Supplementary Figure S1B).

**FIGURE 4:**
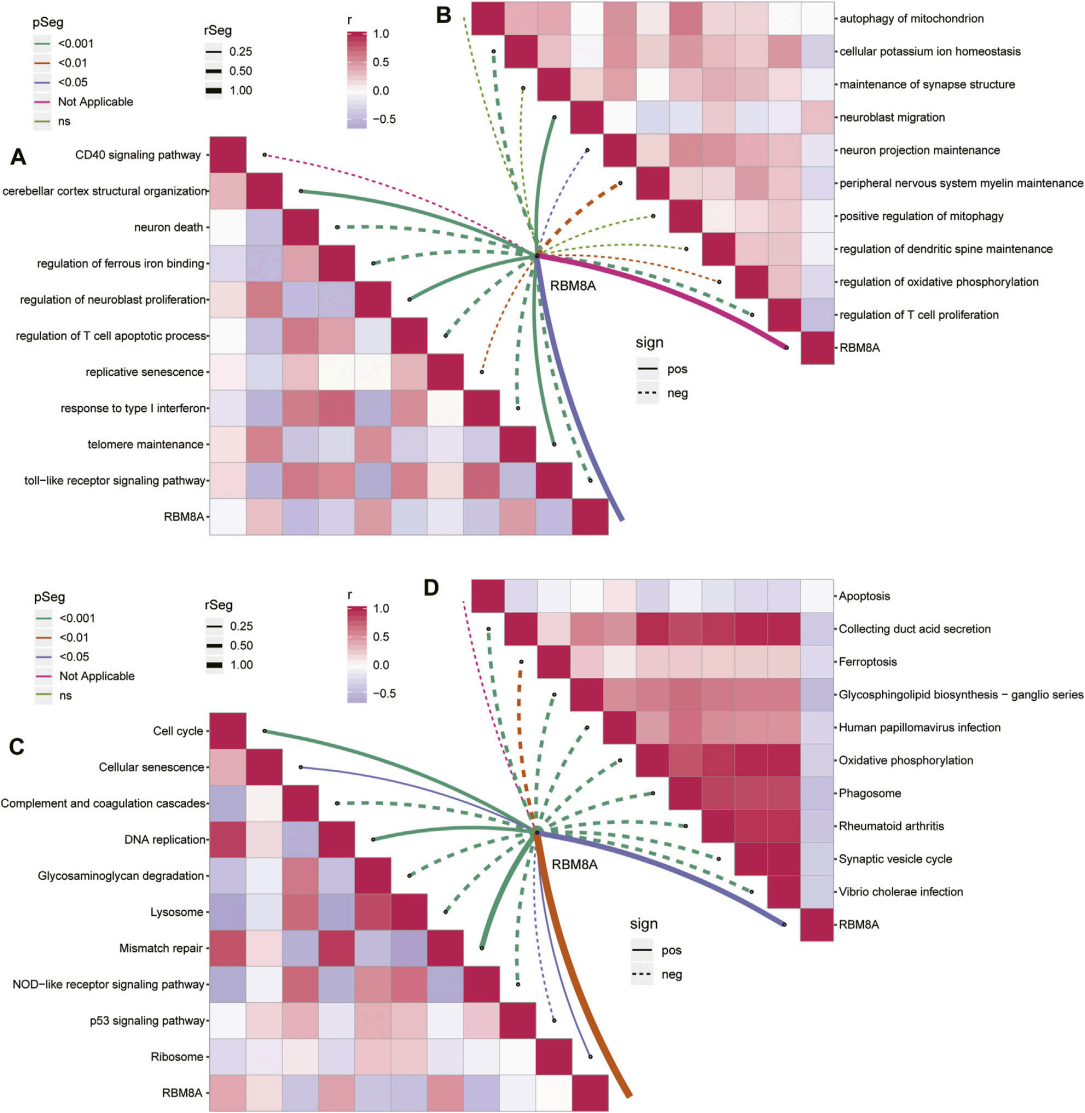
Enrichment analysis of common mRNAs in GBM. **(A,B)**. Correlation between RBM8A and biological processes **(A)** activated or **(B)** inhibited by common mRNAs. **(C,D)**. Correlation between RBM8A and KEGG pathways **(C)** activated or **(B)** inhibited by common mRNAs.

Analysis of the 488 common mRNAs closely related to RBM8A and GBM showed enrichment in activation of neuroblast proliferation and telomere maintenance, but inhibition of T cell apoptosis and response to type I interferon (Figure 4A). Moreover, RBM8A expression correlated positively with inhibition of neuroblast migration but negatively with inhibition of T cell proliferation and cellular potassium ion homeostasis (Figure 4B).

The common mRNAs correlated positively with the KEGG pathways of activated cell cycle and mismatch repair, but negatively with the pathways of activated complement, coagulation, and NOD−like receptor signaling (Figure 4C). RBM8A correlated negatively with inhibition of synaptic vesicle cycling and oxidative phosphorylation (Figure 4D).

### Construction of an RBM8A-Related ceRNA Network

To identify the regulatory network associated with RBM8A, we first screened for DElncRs in TCGA and GSE16011 that were up- or downregulated in both datasets and were associated with patient survival. In the end, we identified nine upregulated lncRNAs: CRNDE, DLEU1, LEF1-AS1, LINC01426, LINC01516, RARA-AS1, SOX21-AS1, TRAF3IP2-AS1, and ZEB1-AS1. We also identified nine downregulated lncRNAs: CYP1B1-AS1, LGALS8-AS1, LINC01616, LINC02347, LINC02361, LINC02433, MATN1-AS1, RBAKDN, and UNC5B-AS1.

**FIGURE 5:**
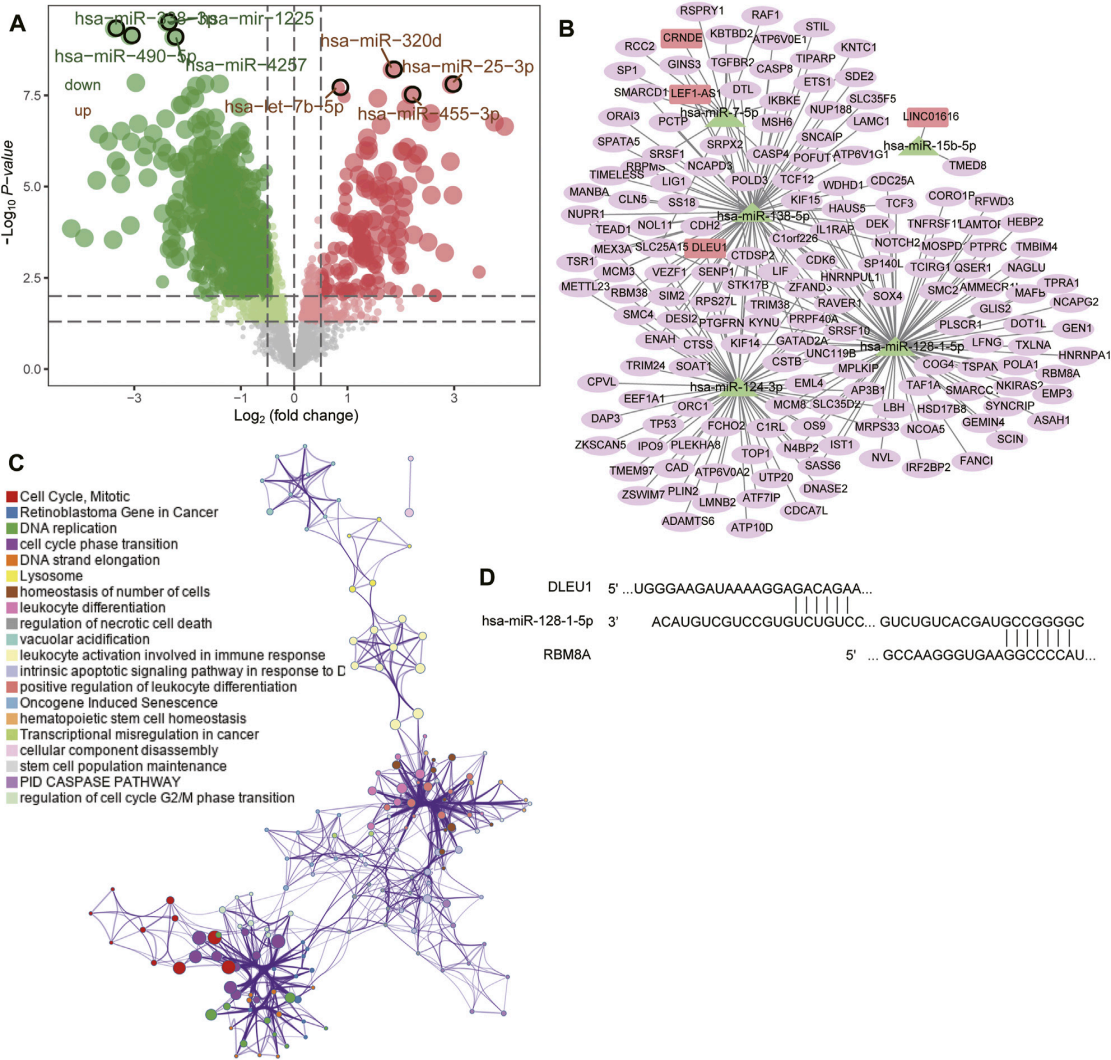
Prediction of target genes and construction of a competing endogenous RNA (ceRNA) network. **(A)** Volcano plot of differentially expressed miRNAs between GBM and controls in GSE90603. Red means upregulated; green, downregulated. **(B)** The ceRNA network in GBM. Rectangles indicate lncRNAs; triangles, miRNAs; and ellipses, mRNAs. **(C)** Functional enrichment analysis of differentially expressed mRNAs in the ceRNA network. **(D)** Base pairing of the 3′-untranslated regions in the DLEU1 and RBM8A mRNAs with has-miR-128-1-5p, as predicted by lncBase 3 and TargetScan, respectively.

Next we predicted the miRNAs targeted by these 18 lncRNAs using the lncBase 3 database. In GSE90603, we identified 1,389 DEmiRs between GBM and controls (Figure 5A), of which five overlapped with the miRNAs targeted by DElncRs: hsa-miR-124-3p, hsa-miR-128-1-5p, hsa-miR-138-5p, hsa-miR-7-5p, and hsa-miR-15b-5p. We focused on these miRNAs because they may be particularly important in GBM. Using the miRWalk and TargetScan databases, we predicted the mRNAs targeted by these five DEmiRs and identified 161 that overlapped with GBM-associated DEmRs.

We synthesized the GBM-associated lncRNAs, miRNAs and mRNAs into a ceRNA network (Figure 5B). Four lncRNAs in the network (CRNDE, DLEU1, LEF1-AS1, and LINC01616) were significantly associated with overall survival of GBM patients (Supplementary Figure S2) and were involved mainly in regulating the cell cycle, mitosis, leukocyte activation during immune responses, and DNA replication (Figure 5C). We found that RBM8A may compete with DLEU1 for binding to hsa-miR-128-1-5p (Figure 5D).

### Identification of RBM8A-Related Candidate mRNAs

**FIGURE 6:**
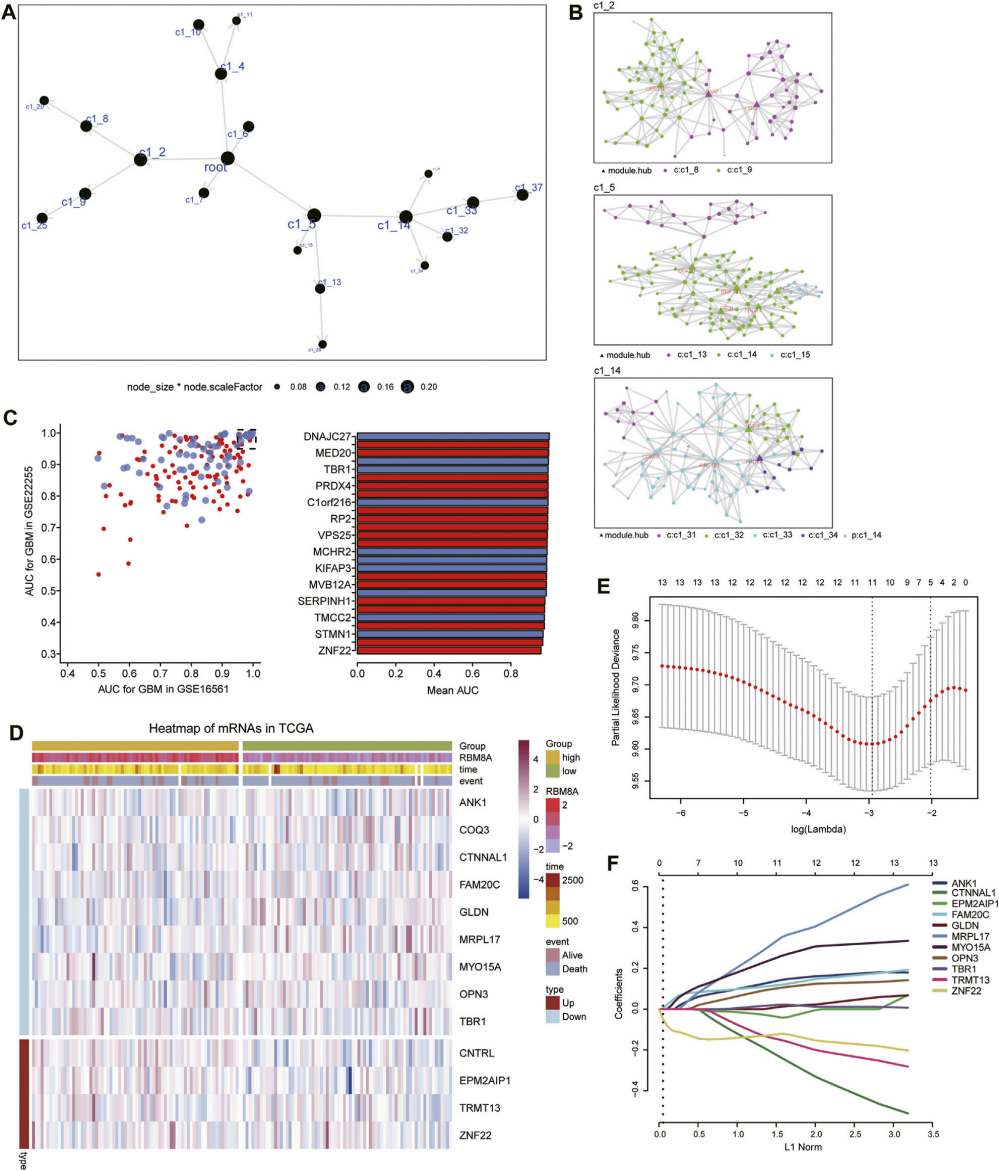
Identification of candidate mRNAs. **(A)** The MEGENA coexpression network of common mRNAs. Nodes represent different modules: the larger the node, the greater the number of module genes. **(B)** Child modules in the MEGENA network featuring the largest number of module genes. Different colors represent different modules, while triangles represent hub genes of modules. **(C)** Module genes with areas under the receiver operating characteristic curve (AUCs) > 0.95 in both TCGA and GSE16011. **(D)** Heatmap of 13 genes related to overall survival in RBM8A^high^ and RBM8A^low^ patients. **(E)** Selection of the optimal lambda parameter *via* minimum criteria in the LASSO regression model. **(F)** LASSO coefficient profiles of 11 genes with non-zero coefficients.

Using MEGENA, we constructed coexpression networks of common mRNAs (Figure 6A). MEGENA identified 37 modules and 336 module genes. Among them, the largest module, C1_5, consisted of 134 genes, while module C1_2 consisted of 96 genes, and C1_14 consisted of 103 genes (Figure 6B). Further, we identified 174 mRNAs whose module genes had AUCs >0.7 in both TCGA and GSE16011 (Figure 6C), of which 13 mRNAs were significantly associated with overall survival of GBM patients (Figure 6D). We assessed 11 of these mRNAs as potential prognostic markers in the LASSO algorithm: ANK1, CTNNAL1, EPM2AIP1, FAM20C, GLDN, MRPL17, MYO15A, OPN3, TBR1, TRMT13, and ZNF22 (Figures 6E,F).

**FIGURE 7:**
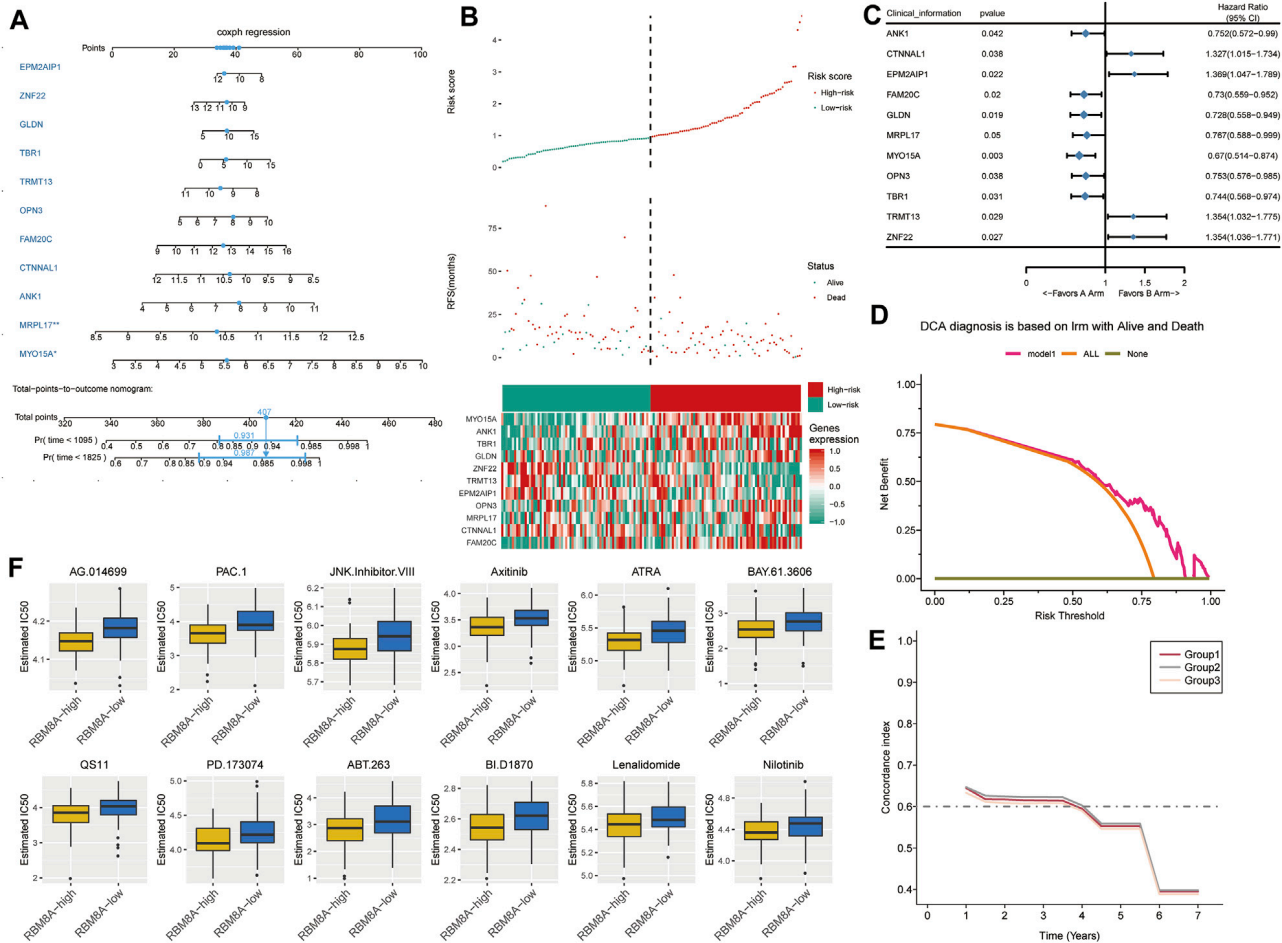
The survival prediction model for GBM based on candidate mRNAs. **(A)** A nomogram prediction model was constructed based on candidate mRNAs in TCGA. **(B)** Distribution of candidate mRNA‐based risk scores, mRNA expression levels, and patient survival in TCGA. **(C)** Forest plots of Cox regression analysis of candidate mRNAs. **(D)** Decision curve analysis of candidate mRNAs for predicting survival status. **(E)** C-index comparison among group 1 (all candidate mRNAs), group 2 [candidate mRNAs with areas under the receiver operating characteristic curve (AUCs) > 0.5] and group 3 (candidate mRNAs with AUCs >0.6). **(F)** Box plots to estimate IC_50_ for chemotherapies to treat RBM8A^high^ and RBM8A^low^ patients.

We used a multivariate Cox model to build a nomogram (Figure 7A). GBM patients were divided into low- or high-risk groups according to the median risk score based on the candidate mRNAs (Figure 7B). High risk scores were associated with greater risk of mortality. High expression and high risk scores for MYO15A, ANK1, TBR1, GLDN, OPN3, MRPL17, and FAM20C emerged as risk factors. Conversely, high expression and low risk scores for ZNF22, TRMT13, EPM2AIP1, and CTNNAL1 emerged as protective factors.

These results were confirmed by using univariate Cox regression to assess the prognostic value of candidate mRNAs (Figure 7C). In fact, decision curve analysis indicated that candidate mRNAs could diagnose GBM better than either the “all-patients-died” scheme or the “no-patients-died” scheme (Figure 7D).

FAM20C, OPN3, TBR1, ANK1, and MYO15A showed AUCs >0.5 for predicting overall survival of GBM patients at 1, 3 and 5 years (Supplementary Figure S3). ANK1 and MYO15A showed AUCs >0.6 for predicting overall survival at the three time points. Candidate mRNAs with AUCs >0.5 showed a higher average concordance index than other DEmRs (Figure 7E).

Next we trained a predictive model against GDSC data in order to estimate the IC_50_ for each sample in the RBM8A^high^ and RBM8A^low^ groups. We predicted 12 drugs that might affect genes in the RBM8A^high^ and RBM8A^low^ groups (Figure 7F): AG.014699, PAC.1, JNK.Inhibitor.VIII, Axitinib, ATRA, BAY.61.3606, QS11, PD.173074, ABT.263, BI.D1870, Lenalidomide, and Nilotinib.

### Correlation Between Immune Infiltration and RBM8A Expression in GBM

**FIGURE 8:**
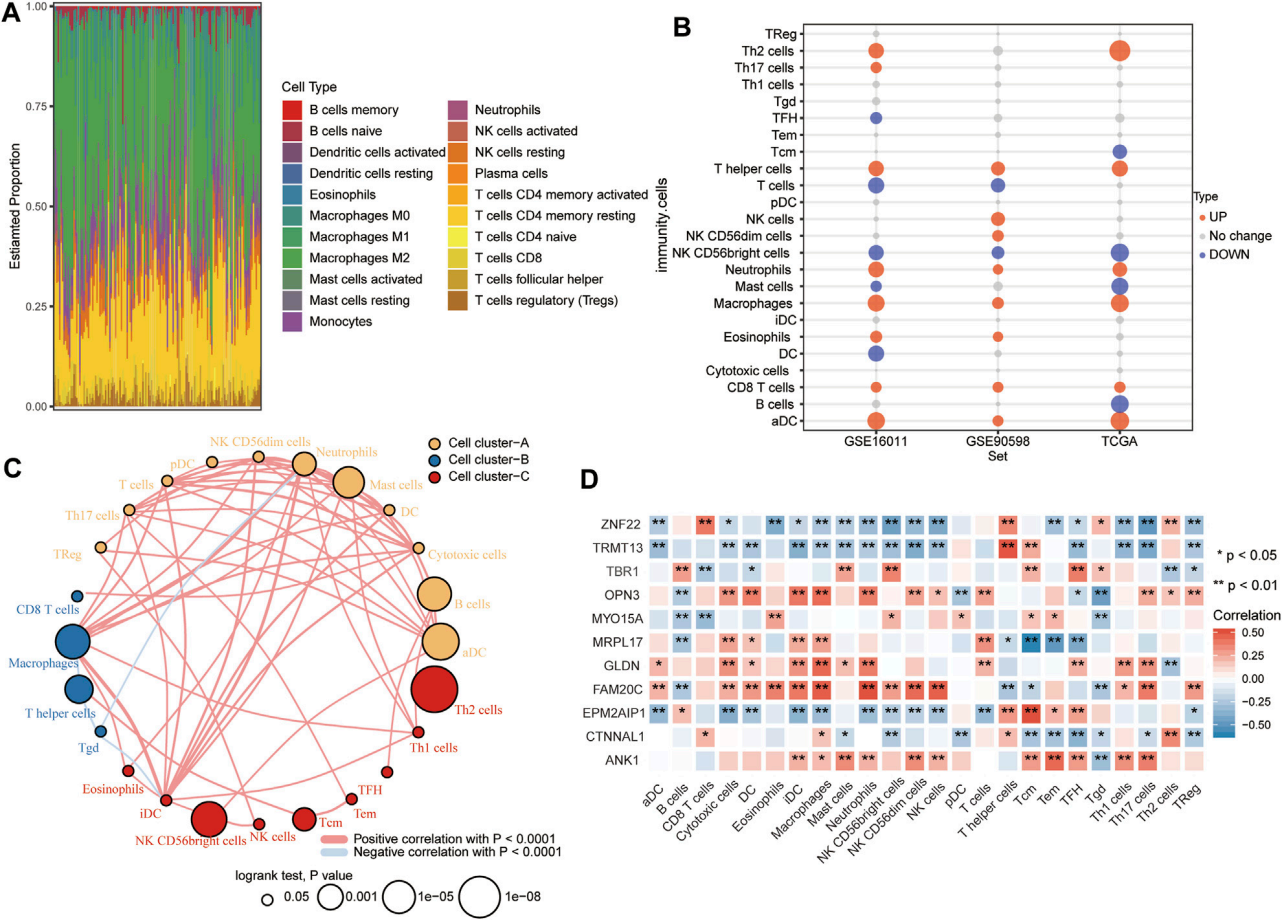
Relationships between RBM8A expression and tumor infiltration by different types of immune cells in GBM. **(A)** Proportions of immune cell types in GBM patients in TCGA, as detected by the CIBERSORT algorithm. **(B)** Levels of infiltration by immune cell types in GBM and controls in TCGA, GSE16011, and GSE90598 datasets. **(C)** Correlation and clustering of tumor-infiltrating immune cell types. **(D)** Correlation between levels of candidate mRNAs and levels of tumor-infiltrating immune cell types. *p < 0.05, **p < 0.01.

According to CIBERSORT, M2 macrophages, CD4^+^ resting memory T cells and neutrophils had larger specific weights among immune cells in GBM samples than in controls in TCGA (Figure 8A). The ssGSEA showed that across all three datasets, levels of infiltration by T helper cells, neutrophils, macrophages, CD8^+^ T cells, and activated dendritic cells (aDC) were significantly higher in GBM than in control samples (Figure 8B). The immune cells fell into three clusters (Figure 8C). The RBM8A^high^ group showed strong infiltration by B cells and Tcm cells, and weak infiltration by macrophages, cytotoxic cells, iDC, T cells, neutrophils, and DC. Levels of candidate mRNAs correlated significantly with levels of immune cell infiltration (Figure 8D).

**FIGURE 9:**
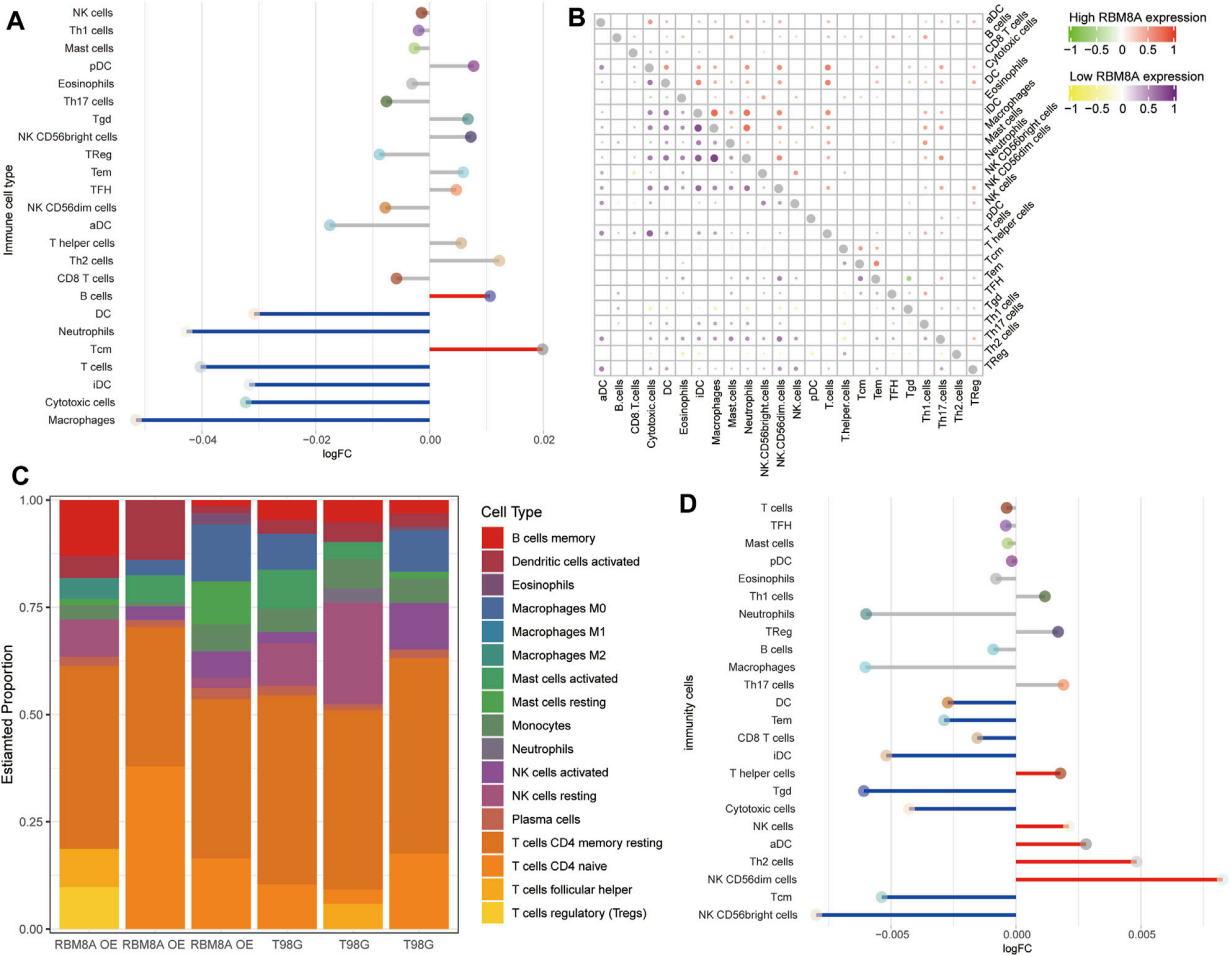
Immune cell infiltration associated with RBM8A expression in GBM. **(A)** Levels of infiltration by 24 immune cell types in RBM8A^high^ and RBM8A^low^ groups in TCGA. **(B)** Correlation of immune cell infiltration between the RBM8A^high^ and RBM8A^low^ groups. **(C)** Proportions of 21 immune cell types in RBM8A-overexpressing T98G cells and controls, as detected by the CIBERSORT algorithm. **(D)** Levels of infiltration by 24 immune cell types in RBM8A-overexpressing and control T98G cells.

To find out more about the role of RBM8A in immune infiltration, we compared the differences in immune infiltration between the RBM8A^high^ and RBM8A^low^ groups (Figure 9A). In both groups, macrophages correlated strongly with neutrophils (Figure 9B). In RBM8A-overexpressing T98G cells, regulatory T cells had larger specific weight among immune cells (Figure 9C), while DC, iDC, and cytotoxic cells showed less infiltration than in control T98G cells (Figure 9D). These results suggest that aberrant RBM8A expression is associated with immune cell infiltration.

### Methylation Modification and Genomic Alterations Associated With Candidate mRNAs in GBM

**FIGURE 10:**
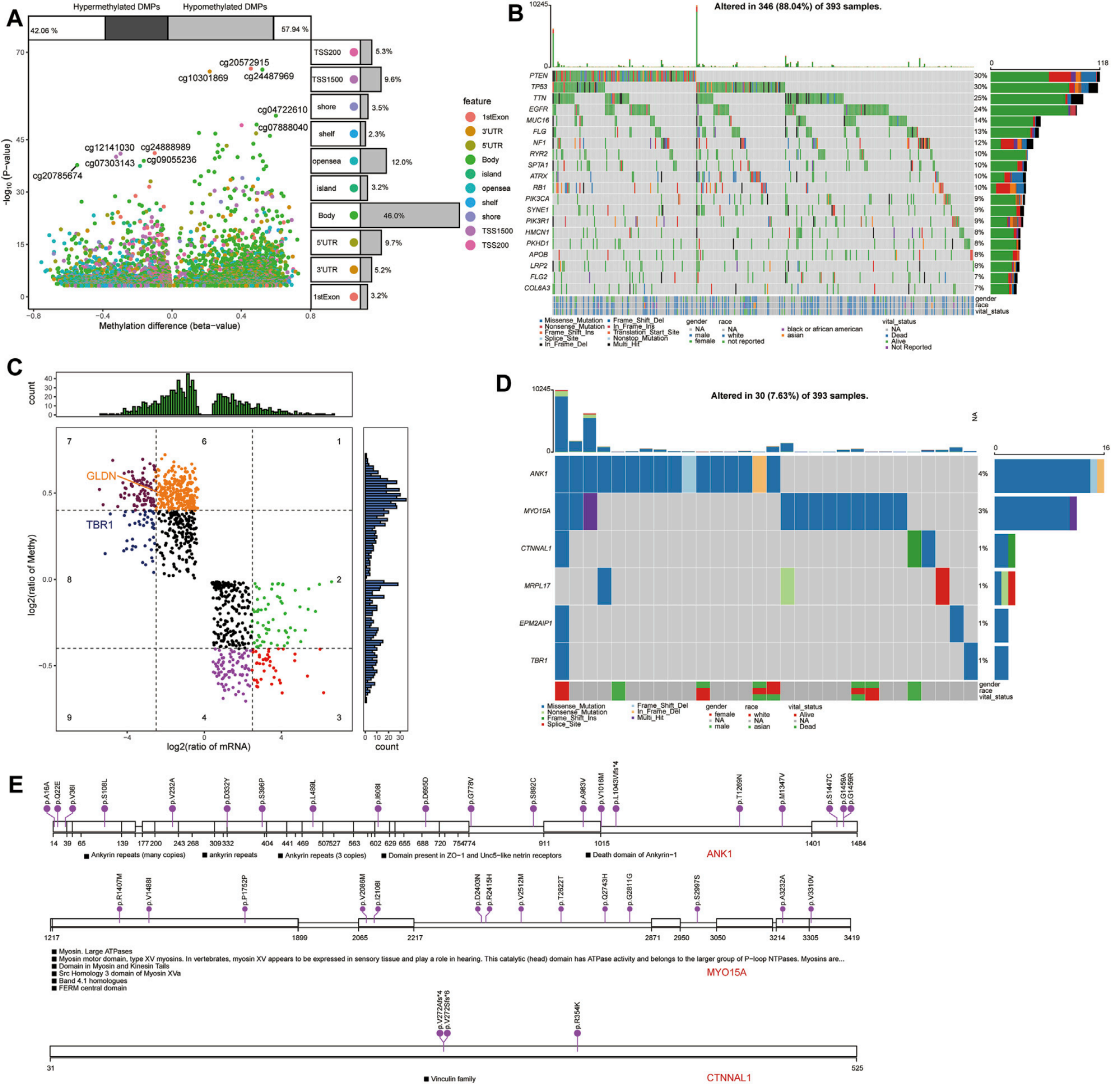
Methylation modification and mutational landscape of candidate mRNAs. **(A)** Differentially methylated probes between GBM and controls in TCGA. DMPs, differentially methylated probes. **(B)** DeltaBeta levels and expression levels of methylation markers. **(C)** Top 20 somatic mutations in GBM in TCGA. **(D)** Mutation spectrum in candidate mRNAs in GBM patients. **(E)** Mutations in the top three candidate mRNAs in GBM.

Comparison of differentially methylated probes (DMPs) between GBM and control samples in TCGA (Figure 10A) allowed us to identify 2,801 upregulated DMPs corresponding to 1,365 genes and 2,033 downregulated DMPs corresponding to 1,705 genes in GBM patients. Among the methylation markers identified in view of opposing methylation levels versus expression levels, GLDN, and TBR1 in candidate mRNAs were subject to methylation modifications (Figure 10B).

Analysis of somatic mutations in GBM samples in TCGA revealed frequent mutations in the *PTEN*, *TP53*, *TTN*, and *EGFR* genes (Figure 10C). We also detected mutations in the candidate mRNAs ANK1, MYO15A, CTNNAL1, MRPL17, EPM2AIP1, and TBR1 in GBM samples (Figures 10D,E).

## Discussion

GBM is the most common and aggressive primary brain tumor. Intensive multimodal treatments involving surgery, radiotherapy and chemotherapy have failed to substantially improve median survival (Hassn Mesrati et al., 2020). Elucidating the molecular mechanisms of GBM pathogenesis and finding promising therapeutic targets are essential to improve patient outcomes. Therefore, in this study, we sought to gain a more detailed understanding of the RBM8A-associated regulatory networks and molecular pathways that may affect prognosis. Our results strongly suggest that RBM8A is an important oncogene and may help identify related molecules that could be useful therapeutic targets.

RBM8A is a core component of the exon junction complex (EJC), through which it contributes to the formation and development of the nervous system (McSweeney et al., 2020). RBM8A deficiency leads to muscle disorganization, neuronal cell death, and motor neuron growth defects (Gangras et al., 2020); conversely, increased RBM8A expression leads to increased proliferation of cells in the ventricular and subventricular zones, while decreasing the migration of cells toward the cortical plate (Zou et al., 2015). Differential expression and dysfunction of EJC core proteins have been reported in multiple cancers (Degot et al., 2004; Kahles et al., 2018; Liu et al., 2020). The results of our analysis show that RBM8A is highly expressed in GBM and positively correlates with tumor purity. Our work provides the first evidence that RBM8A promotes GBM.

In particular, our enrichment analysis linked RBM8A to neuroblast proliferation, telomere maintenance, T cell apoptotic process, and type I interferon in GBM (Mahata et al., 2015; Wang et al., 2016; Feuerbach et al., 2019; Hu et al., 2019). Higher RBM8A expression and GBM appear to be associated with inhibition of neuroblast migration and T cell proliferation. Similarly, RBM8A expression appears to be linked to complement activation, which can promote tumor growth and metastasis (Afshar-Kharghan, 2017). Coagulation factors or their downstream targets can lead to pathophysiology of the central nervous system (De Luca et al., 2017). Dysregulation of NOD-like receptor signaling has been implicated in microbial infection, diabetes, cardiac and metabolic disorders, autoimmune diseases, and cancer (Davis et al., 2011). Indeed, NOD-like receptors may be markers of glioma progression (Sharma et al., 2019). Our previous study confirmed that RBM8A overexpression promoted glioblastoma growth and invasion through the Notch/STAT3 pathway (Lin et al., 2021). The metabolic pathway of oxidative phosphorylation may be a therapeutic target against GBM (Shi et al., 2019; Szmidt et al., 2019).

Our RBM8A-associated ceRNA network suggests that RBM8A competes with DLEU1 for binding to hsa-miR-128-1-5p. DLEU1 has been implicated in the development and progression of several cancers, including GBM (Feng et al., 2019). DLEU1 is upregulated in GBM, and its knockout inhibits GBM cell proliferation and apoptosis, while sensitizing tumor cells to temozolomide (Liu et al., 2019; Lv et al., 2020). Conversely, upregulating hsa-miR-128-1-5p inhibits glioma cell proliferation and promotes apoptosis (Dahai et al., 2020), and the same miRNA has been implicated in hepatocellular carcinoma (Xu et al., 2021). The ceRNA network developed here may help guide future studies of how RBM8A contributes to GBM.

We found that RBM8A-related candidate mRNAs were strongly associated with prognosis of GBM patients, especially ANK1 and MYO15A. These results echo previous studies linking these two mRNAs to GBM prognosis (Serão et al., 2011; Li and Meng, 2021). Methylation of the ANK1 gene changes during development, and this is important for regulating formation of the nervous system (Gasparoni et al., 2018). ANK1 is also involved in TGF-β/Smad and Wnt/β-Catenin signaling, both of which are associated with poor overall survival in GBM patients (Hallal et al., 2020). MYO15A helps define cellular morphology and has been associated with neurological disorders (Hicks et al., 2020).

We found that tumor tissues of GBM patients expressing high levels of RBM8A show substantial infiltration by B cells and central memory T cells. This may reflect that GBM is particularly adopt at subverting antitumor immunity, leading to profound T cell dysfunction (Woroniecka et al., 2018). GBM can be treated effectively by using immunotherapy to sharpen immune responses against tumors (Wang et al., 2019; Land et al., 2020). Our results may help identify specific T cell subsets and signaling pathways as new therapeutic targets. In addition, our work identified alterations in DNA methylation and somatic mutations that suggest the possibility of additional therapeutic targets that should be explored.

Nevertheless, our study does have some limitations. First, the RBM8A-related risk score model requires further validation in multicenter clinical trials and prospective studies. Indeed, the candidate mRNAs should be verified and analyzed in detail in cellular and biochemical studies. In fact, the network of lncRNAs, miRNAs, and mRNAs described here should be explored in order to establish the full range of processes through which RBM8A may influence GBM onset and progression. In addition, there are some limitations in this study. The role of RBM8A on chemotherapy resistance has not been addressed in current study, which will be pursued in future studies. The hedgehog, and JAK−STAT pathways need to be supported by functional studies in future.

## Conclusion

We found that RBM8A may be a diagnostic marker, oncogene and therapeutic target in GBM. We established and validated a risk score model based on 11 candidate mRNAs that can reliably predict overall survival of GBM patients. This work may move us closer to individualized treatment and management of GBM as well as more detailed understanding of its pathology.

## Data Availability

The original contributions presented in the study are included in the article/Supplementary Material, further inquiries can be directed to the corresponding authors.

## Supplementary Material

Supplementary Figure S1
**(A)**. GSEAcomparing GBM and controls. **(B)**. GSEA comparing RBM8A^high^ and RBM8A^low^ groups.

Supplementary Figure S2 Association of long noncoding RNAs in the ceRNA network with overall survival of GBM patients.

Supplementary Figure S3 Ability of candidate mRNAs to predict overall survival at one, three or five years, based on the area under receiver operating characteristic curves (AUC). Only mRNAs showing AUCs >0.6 in TCGA are shown.

## Abbreviations

AUC, Area under the receiver operating characteristic curve; BP, Biological process; C-index, Concordance index; DCA, Decision curve analyses; DElncRs, Differentially expressed lncRNAs; DEmRs, Differentially expressed mRNAs; DEmiRs, Differentially expressed miRNAs; DLEU1, Lymphocytic leukemia 1; DMPs, Differentially methylated probes; EJC, Exon junction complex; GBM, Glioblastoma; GDSC, Genomics of drug sensitivity in cancer; GEO, Gene Expression Omnibus; GO, Gene Ontology; GSEA, gene set enrichment analysis; GSVA, Gene set variation analysis; KEGG, Kyoto Encyclopedia of Genes and Genomes; K-M, Kaplan-Meier; LASSO, Least absolute shrinkage and selection operator regression; MEGENA, Multiscale embedded gene coexpression network analysis; OE, Overexpressing; OS, overall survival; PFN, planar filtered network; RBM, RNA binding motif protein; RBM8A, RNA-binding motif protein 8A; TCGA, The Cancer Genome Atlas; Tcm, Memory T cell; Tregs, T cells regulatory.

## References

[B1] Afshar-Kharghan V. (2017). The Role of the Complement System in Cancer. J. Clin. Invest. 127 (3), 780–789. 10.1172/jci90962 28248200 PMC5330758

[B2] Dahai Z. Daliang C. Famu L. Xiang W. Lenian L. Jianmin C. (2020). Lowly Expressed lncRNA PVT1 Suppresses Proliferation and Advances Apoptosis of Glioma Cells through Up-Regulating microRNA-128-1-5p and Inhibiting PTBP1. Brain Res. Bull. 163, 1–13. 10.1016/j.brainresbull.2020.06.006 32562719

[B3] Davis B. K. Wen H. Ting J. P.-Y. (2011). The Inflammasome NLRs in Immunity, Inflammation, and Associated Diseases. Annu. Rev. Immunol. 29, 707–735. 10.1146/annurev-immunol-031210-101405 21219188 PMC4067317

[B4] De Luca C. Virtuoso A. Maggio N. Papa M. (2017). Neuro-Coagulopathy: Blood Coagulation Factors in Central Nervous System Diseases. Int. J. Mol. Sci. 18 (10), 2128. 10.3390/ijms18102128 PMC5666810 29023416

[B5] Degot S. Le Hir H. Alpy F. Kedinger V. Stoll I. Wendling C. (2004). Association of the Breast Cancer Protein MLN51 with the Exon junction Complex via its Speckle Localizer and RNA Binding Module. J. Biol. Chem. 279 (32), 33702–33715. 10.1074/jbc.m402754200 15166247

[B6] Fathi Kazerooni A. Bakas S. Saligheh Rad H. Davatzikos C. (2020). Imaging Signatures of Glioblastoma Molecular Characteristics: A Radiogenomics Review. J. Magn. Reson. Imaging 52 (1), 54–69. 10.1002/jmri.26907 31456318 PMC7457548

[B7] Feng L. He M. Rao M. Diao J. Zhu Y. (2019). Long Noncoding RNA DLEU1 Aggravates Glioma Progression via the miR-421/MEF2D axis. Ott Vol. 12, 5405–5414. 10.2147/ott.s207542 PMC6625645 31360066

[B8] Feuerbach L. Sieverling L. Deeg K. I. Ginsbach P. Hutter B. Buchhalter I. (2019). TelomereHunter - In Silico Estimation of Telomere Content and Composition from Cancer Genomes. BMC Bioinformatics 20 (1), 272. 10.1186/s12859-019-2851-0 31138115 PMC6540518

[B9] Friedman J. Hastie T. Tibshirani R. (2010). Regularization Paths for Generalized Linear Models via Coordinate Descent. J. Stat. Softw. 33 (1), 1–22. 10.18637/jss.v033.i01 20808728 PMC2929880

[B10] Gangras P. Gallagher T. L. Parthun M. A. Yi Z. Patton R. D. Tietz K. T. (2020). Zebrafish Rbm8a and Magoh Mutants Reveal EJC Developmental Functions and New 3′UTR Intron-Containing NMD Targets. Plos Genet. 16 (6), e1008830. 10.1371/journal.pgen.1008830 32502192 PMC7310861

[B11] Gasparoni G. Bultmann S. Lutsik P. Kraus T. F. J. Sordon S. Vlcek J. (2018). DNA Methylation Analysis on Purified Neurons and Glia Dissects Age and Alzheimer's Disease-specific Changes in the Human Cortex. Epigenetics & Chromatin 11 (1), 41. 10.1186/s13072-018-0211-3 30045751 PMC6058387

[B12] Hallal S. Ebrahim Khani S. Wei H. Lee M. Y. T. Sim H. W. Sy J. (2020). Deep Sequencing of Small RNAs from Neurosurgical Extracellular Vesicles Substantiates miR-486-3p as a Circulating Biomarker that Distinguishes Glioblastoma from Lower-Grade Astrocytoma Patients. Int. J. Mol. Sci. 21 (14), 4954. 10.3390/ijms21144954 PMC7404297 32668808

[B13] Hänzelmann S. Castelo R. Guinney J. (2013). GSVA: Gene Set Variation Analysis for Microarray and RNA-Seq Data. BMC Bioinformatics 14, 7. 10.1186/1471-2105-14-7 23323831 PMC3618321

[B14] Hassn Mesrati M. Behrooz A. B. Abuhamad A. Y. Syahir A. (2020). Understanding Glioblastoma Biomarkers: Knocking a Mountain with a Hammer. Cells 9 (5), 1236. 10.3390/cells9051236 PMC7291262 32429463

[B15] Hicks D. A. Jones A. C. Corbett N. J. Fisher K. Pickering-Brown S. M. Ashe M. P. (2020). Extracellular Vesicles Isolated from Human Induced Pluripotent Stem Cell-Derived Neurons Contain a Transcriptional Network. Neurochem. Res. 45 (7), 1711–1728. 10.1007/s11064-020-03019-w 32361798 PMC7297870

[B16] Hu H. J. Wang S. S. Wang Y. X. Liu Y. Feng X. M. Shen Y. (2019). Blockade of the Forward Na +/Ca 2+ Exchanger Suppresses the Growth of Glioblastoma Cells through Ca 2+ ‐mediated Cell Death. Br. J. Pharmacol. 176 (15), 2691–2707. 10.1111/bph.14692 31034096 PMC6609550

[B17] Ishigaki Y. Nakamura Y. Tatsuno T. Hashimoto M. Shimasaki T. Iwabuchi K. (2013). Depletion of RNA-Binding Protein RBM8A (Y14) Causes Cell Cycle Deficiency and Apoptosis in Human Cells. Exp. Biol. Med. (Maywood) 238 (8), 889–897. 10.1177/1535370213494646 23970407

[B18] Kahles A. Lehmann K. V. Toussaint N. C. Hüser M. Stark S. G. Sachsenberg T. (2018). Comprehensive Analysis of Alternative Splicing across Tumors from 8,705 Patients. Cancer Cell 34 (2), 211–e6. 10.1016/j.ccell.2018.07.001 30078747 PMC9844097

[B19] Land C. A. Musich P. R. Haydar D. Krenciute G. Xie Q. (2020). Chimeric Antigen Receptor T-Cell Therapy in Glioblastoma: Charging the T Cells to Fight. J. Transl Med. 18 (1), 428. 10.1186/s12967-020-02598-0 33176788 PMC7659102

[B20] Lau D. Magill S. T. Aghi M. K. (2014). Molecularly Targeted Therapies for Recurrent Glioblastoma: Current and Future Targets. Foc 37 (6), E15. 10.3171/2014.9.focus14519 PMC5058366 25434384

[B21] Li X. Meng Y. (2021). SUMOylation Regulator-Related Molecules Can Be Used as Prognostic Biomarkers for Glioblastoma. Front. Cel Dev. Biol. 9, 658856. 10.3389/fcell.2021.658856 PMC8063029 33898460

[B22] Liang R. Zhang J. Liu Z. Liu Z. Li Q. Luo X. (2020). Mechanism and Molecular Network of RBM8A-Mediated Regulation of Oxaliplatin Resistance in Hepatocellular Carcinoma. Front. Oncol. 10, 585452. 10.3389/fonc.2020.585452 33552961 PMC7862710

[B23] Lin Y. Liang R. Qiu Y. Lv Y. Zhang J. Qin G. (2019). Expression and Gene Regulation Network of RBM8A in Hepatocellular Carcinoma Based on Data Mining. Aging 11 (2), 423–447. 10.18632/aging.101749 30670676 PMC6366983

[B24] Lin Y. Wei L. Hu B. Zhang J. Wei J. Qian Z. (2021). RBM8A Promotes Glioblastoma Growth and Invasion through the Notch/STAT3 Pathway. Front. Oncol. 11, 736941. 10.3389/fonc.2021.736941 34804926 PMC8600138

[B25] Liu X. Chen R. Liu L. (2019). SP1-DLEU1-miR-4429 Feedback Loop Promotes Cell Proliferative and Anti-apoptotic Abilities in Human Glioblastoma. Biosci. Rep. 39 (12), BSR20190994. 10.1042/BSR20190994 31713587 PMC6900472

[B26] Liu Y. Yang Y. Luo Y. Wang J. Lu X. Yang Z. (2020). Prognostic Potential of PRPF3 in Hepatocellular Carcinoma. Aging 12 (1), 912–930. 10.18632/aging.102665 31926109 PMC6977647

[B27] Love M. I. Huber W. Anders S. (2014). Moderated Estimation of Fold Change and Dispersion for RNA-Seq Data with DESeq2. Genome Biol. 15 (12), 550. 10.1186/s13059-014-0550-8 25516281 PMC4302049

[B28] Lu C.-C. Lee C.-C. Tseng C.-T. Tarn W.-Y. (2017). Y14 Governs P53 Expression and Modulates DNA Damage Sensitivity. Sci. Rep. 7, 45558. 10.1038/srep45558 28361991 PMC5374521

[B29] Lv Q.-L. Wang L.-C. Li D.-C. Lin Q.-X. Shen X.-L. Liu H.-Y. (2020). Knockdown lncRNA DLEU1 Inhibits Gliomas Progression and Promotes Temozolomide Chemosensitivity by Regulating Autophagy. Front. Pharmacol. 11, 560543. 10.3389/fphar.2020.560543 33362537 PMC7756250

[B30] Lv X. Cheng H. (2020). Prognostic Value of Increased Expression of RBM8A in Gastric Cancer. Braz. J. Med. Biol. Res. 53 (4), e9290. 10.1590/1414-431X20209290 32294703 PMC7164227

[B31] Mahata B. Biswas S. Rayman P. Chahlavi A. Ko J. Bhattacharjee A. (2015). GBM Derived Gangliosides Induce T Cell Apoptosis through Activation of the Caspase Cascade Involving Both the Extrinsic and the Intrinsic Pathway. PLoS One 10 (7), e0134425. 10.1371/journal.pone.0134425 26226135 PMC4520498

[B32] Mayakonda A. Lin D.-C. Assenov Y. Plass C. Koeffler H. P. (2018). Maftools: Efficient and Comprehensive Analysis of Somatic Variants in Cancer. Genome Res. 28 (11), 1747–1756. 10.1101/gr.239244.118 30341162 PMC6211645

[B33] McSweeney C. Dong F. Chen M. Vitale J. Xu L. Crowley N. (2020). Full Function of Exon junction Complex Factor, Rbm8a, Is Critical for Interneuron Development. Transl Psychiatry 10 (1), 379. 10.1038/s41398-020-01065-0 33154347 PMC7644723

[B34] Novak M. Koprivnikar Krajnc M. Hrastar B. Breznik B. Majc B. Mlinar M. (2020). CCR5-Mediated Signaling Is Involved in Invasion of Glioblastoma Cells in its Microenvironment. Int. J. Mol. Sci. 21 (12), 4199. 10.3390/ijms21124199 PMC7352708 32545571

[B35] Parker N. R. Khong P. Parkinson J. F. Howell V. M. Wheeler H. R. (2015). Molecular Heterogeneity in Glioblastoma: Potential Clinical Implications. Front. Oncol. 5, 55. 10.3389/fonc.2015.00055 25785247 PMC4347445

[B36] Ritchie M. E. Phipson B. Wu D. Hu Y. Law C. W. Shi W. (2015). Limma powers Differential Expression Analyses for RNA-Sequencing and Microarray Studies. Nucleic Acids Res. 43 (7), e47. 10.1093/nar/gkv007 25605792 PMC4402510

[B37] Robin X. Turck N. Hainard A. Tiberti N. Lisacek F. Sanchez J.-C. (2011). pROC: an Open-Source Package for R and S+ to Analyze and Compare ROC Curves. BMC Bioinformatics 12, 77. 10.1186/1471-2105-12-77 21414208 PMC3068975

[B38] Serão N. V. L. Delfino K. R. Southey B. R. Beever J. E. Rodriguez-Zas S. L. (2011). Cell Cycle and Aging, Morphogenesis, and Response to Stimuli Genes Are Individualized Biomarkers of Glioblastoma Progression and Survival. BMC Med. Genomics 4, 49. 10.1186/1755-8794-4-49 21649900 PMC3127972

[B39] Sharma N. Saxena S. Agrawal I. Singh S. Srinivasan V. Arvind S. (2019). Differential Expression Profile of NLRs and AIM2 in Glioma and Implications for NLRP12 in Glioblastoma. Sci. Rep. 9 (1), 8480. 10.1038/s41598-019-44854-4 31186453 PMC6559951

[B40] Shi Y. Lim S. K. Liang Q. Iyer S. V. Wang H.-Y. Wang Z. (2019). Gboxin Is an Oxidative Phosphorylation Inhibitor that Targets Glioblastoma. Nature 567 (7748), 341–346. 10.1038/s41586-019-0993-x 30842654 PMC6655586

[B41] Song W.-M. Zhang B. (2015). Multiscale Embedded Gene Co-expression Network Analysis. Plos Comput. Biol. 11 (11), e1004574. 10.1371/journal.pcbi.1004574 26618778 PMC4664553

[B42] Subramanian A. Tamayo P. Mootha V. K. Mukherjee S. Ebert B. L. Gillette M. A. (2005). Gene Set Enrichment Analysis: a Knowledge-Based Approach for Interpreting Genome-wide Expression Profiles. Proc. Natl. Acad. Sci. U.S.A. 102 (43), 15545–15550. 10.1073/pnas.0506580102 16199517 PMC1239896

[B43] Szmidt M. Stankiewicz A. Urbańska K. Jaworski S. Kutwin M. Wierzbicki M. (2019). Graphene Oxide Down-Regulates Genes of the Oxidative Phosphorylation Complexes in a Glioblastoma. BMC Mol. Biol 20 (1), 2. 10.1186/s12867-018-0119-2 30602369 PMC6317254

[B44] Tatsuno T. Ishigaki Y. (2018). C-terminal Short Arginine/serine Repeat Sequence-dependent Regulation of Y14 (RBM8A) Localization. Sci. Rep. 8 (1), 612. 10.1038/s41598-017-18765-1 29330450 PMC5766523

[B45] Wang R. Davidoff A. M. Pfeffer L. M. (2016). Bortezomib Sensitizes Human Glioblastoma Cells to Induction of Apoptosis by Type I Interferons through NOXA Expression and Mcl-1 Cleavage. Biochem. Biophysical Res. Commun. 478 (1), 128–134. 10.1016/j.bbrc.2016.07.080 PMC4991636 27450810

[B46] Wang X. Guo G. Guan H. Yu Y. Lu J. Yu J. (2019). Challenges and Potential of PD-1/pd-L1 Checkpoint Blockade Immunotherapy for Glioblastoma. J. Exp. Clin. Cancer Res. 38 (1), 87. 10.1186/s13046-019-1085-3 30777100 PMC6380009

[B47] Woroniecka K. I. Rhodin K. E. Chongsathidkiet P. Keith K. A. Fecci P. E. (2018). T-cell Dysfunction in Glioblastoma: Applying a New Framework. Clin. Cancer Res. 24 (16), 3792–3802. 10.1158/1078-0432.ccr-18-0047 29593027 PMC6095741

[B48] Xie Z. Wu Y. Huang Y. Cai Y. Wang Y. Wang Y. (2021). Prognostic Value and Oncogenic Effect of Increased RBM8A Expression in colon Adenocarcinoma. Biochem. Cel Biol 99 (5), x. 10.1139/bcb-2020-0591 33843263

[B49] Xu F. Jiang L. Zhao Q. Zhang Z. Liu Y. Yang S. (2021). Whole-transcriptome and Proteome Analyses Identify Key Differentially Expressed mRNAs, miRNAs, lncRNAs and circRNAs Associated with HCC. Oncogene 40, 4820–4831. 10.1038/s41388-021-01908-0 34155346

[B50] Xu Y. Geng R. Yuan F. Sun Q. Liu B. Chen Q. (2019). Identification of Differentially Expressed Key Genes between Glioblastoma and Low-Grade Glioma by Bioinformatics Analysis. PeerJ 7, e6560. 10.7717/peerj.6560 30867991 PMC6409090

[B51] Young J. S. Prados M. D. Butowski N. (2018). Using Genomics to Guide Treatment for Glioblastoma. Pharmacogenomics 19 (15), 1217–1229. 10.2217/pgs-2018-0078 30203716

[B52] Yu G. Wang L.-G. Han Y. He Q.-Y. (2012). clusterProfiler: an R Package for Comparing Biological Themes Among Gene Clusters. OMICS: A J. Integr. Biol. 16 (5), 284–287. 10.1089/omi.2011.0118 PMC3339379 22455463

[B53] Zhang Y. Shen B. Zhang D. Wang Y. Tang Z. Ni N. (2017). miR-29a Regulates the Proliferation and Differentiation of Retinal Progenitors by Targeting Rbm8a. Oncotarget 8 (19), 31993–32008. 10.18632/oncotarget.16669 28404883 PMC5458264

[B54] Zhao Y. Huang W. Kim T.-M. Jung Y. Menon L. G. Xing H. (2019). MicroRNA-29a Activates a Multi-Component Growth and Invasion Program in Glioblastoma. J. Exp. Clin. Cancer Res. 38 (1), 36. 10.1186/s13046-019-1026-1 30683134 PMC6347789

[B55] Zou D. Li R. Huang X. Chen G. Liu Y. Meng Y. (2019). Identification of Molecular Correlations of RBM8A with Autophagy in Alzheimer's Disease. Aging 11 (23), 11673–11685. 10.18632/aging.102571 31816601 PMC6932873

[B56] Zou D. McSweeney C. Sebastian A. Reynolds D. J. Dong F. Zhou Y. (2015). A Critical Role of RBM8a in Proliferation and Differentiation of Embryonic Neural Progenitors. Neural Dev. 10, 18. 10.1186/s13064-015-0045-7 26094033 PMC4479087

