# Supplementary figures and images for: Molecular Insights and Prognosis Associated With RBM8A in Glioblastoma
Source: Front Mol Biosci. 2022 Apr 29;9:876603. doi: 10.3389/fmolb.2022.876603 (PMC9098818; doi:10.3389/fmolb.2022.876603)

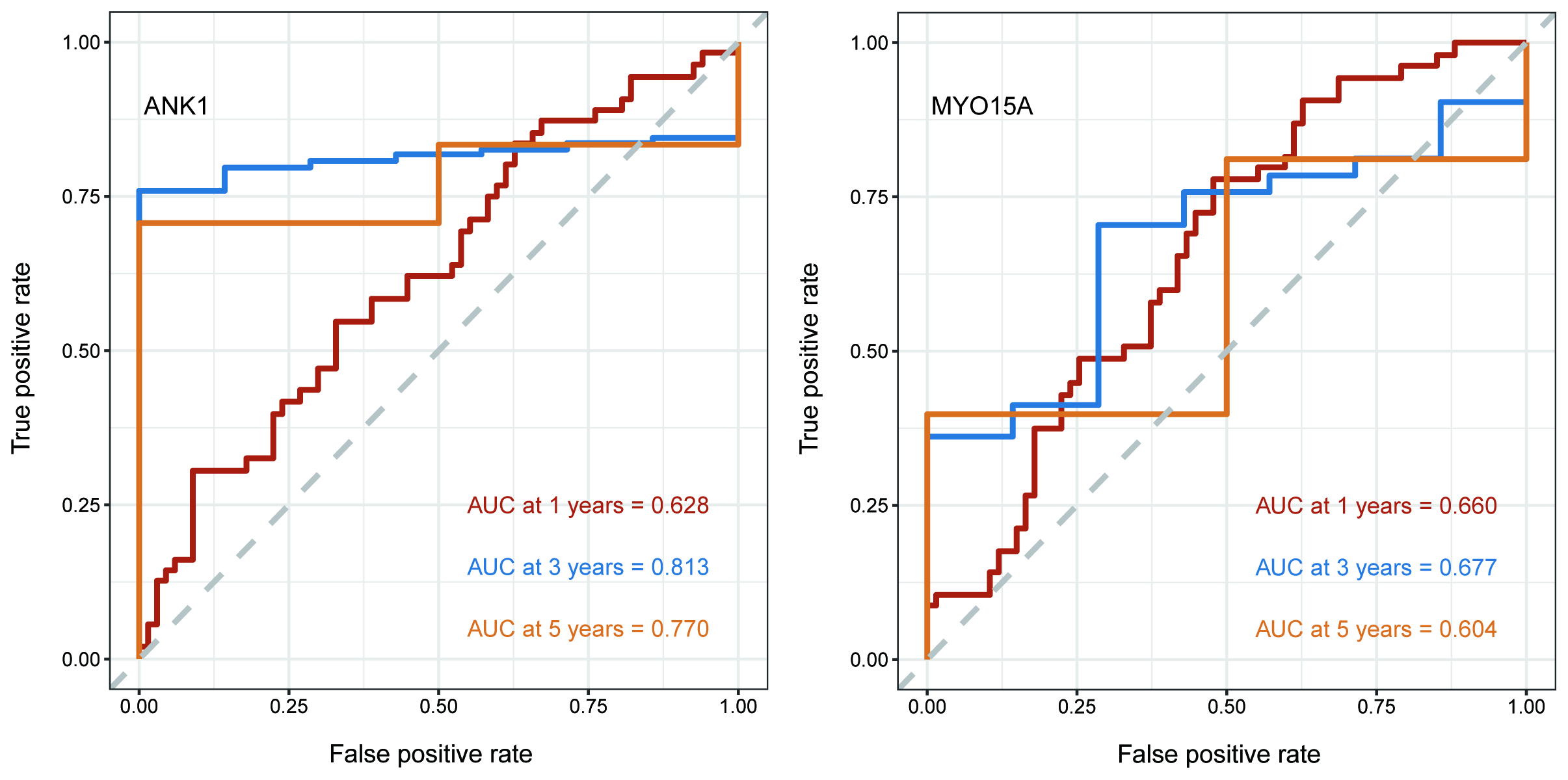

Supplement: Supplementary file 1 [file Image3.TIF]

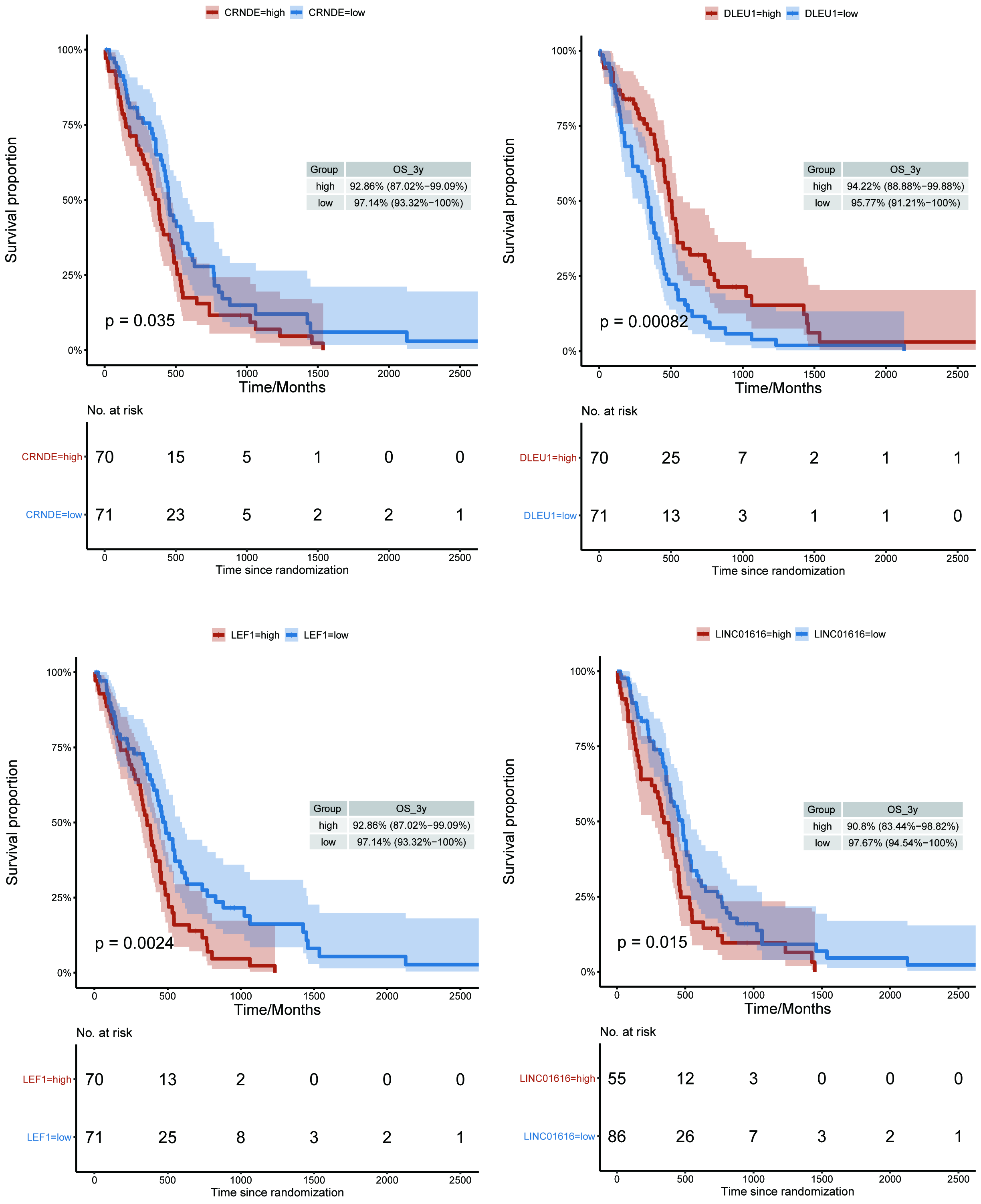

Supplement: Supplementary file 2 [file Image2.tif]

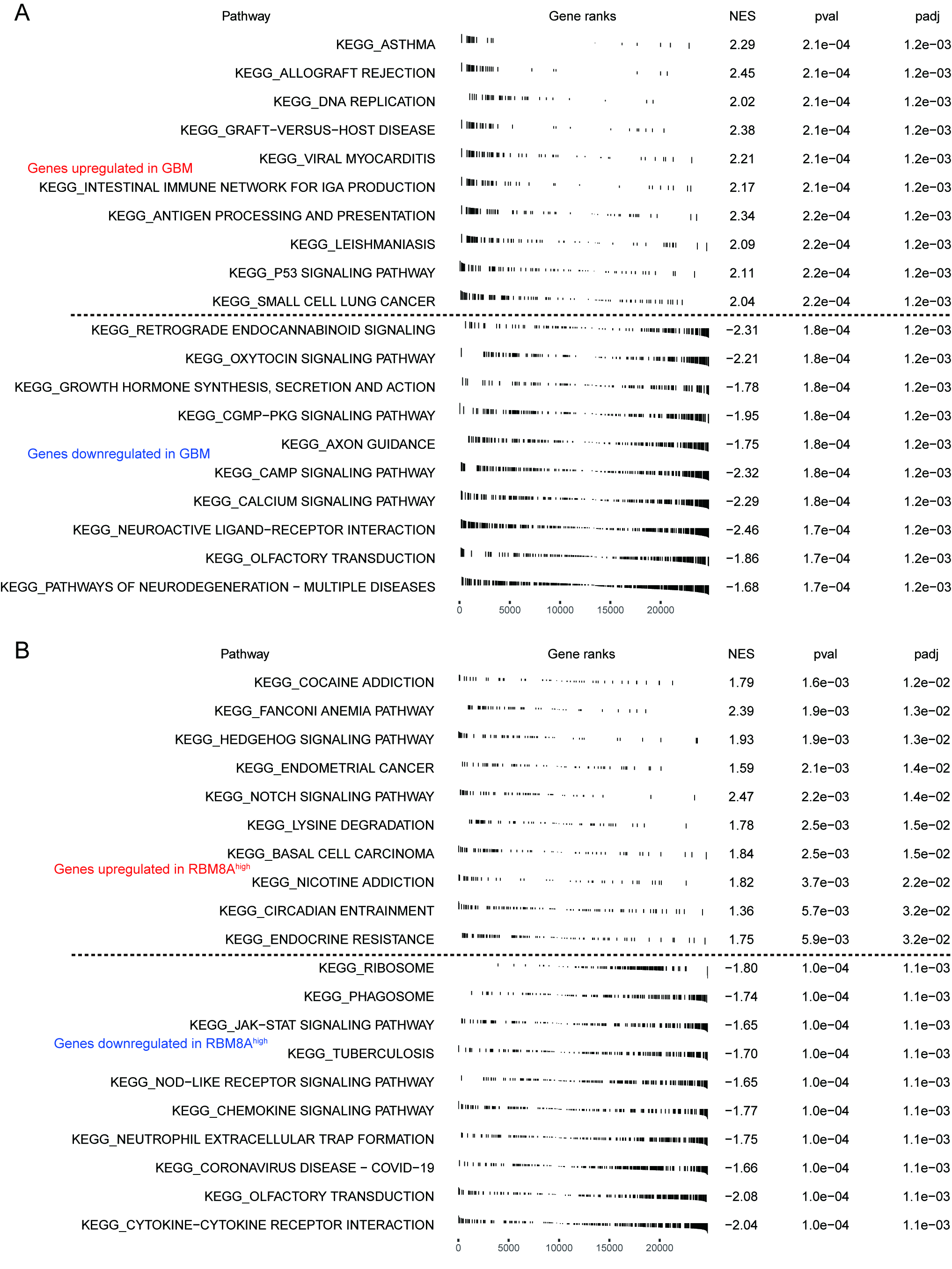

Supplement: Supplementary file 3 [file Image1.TIF]
